# Cheap talk communication with dynamic information searching

**DOI:** 10.1186/s40064-016-2332-0

**Published:** 2016-06-13

**Authors:** Yongjie Zhang, Yuechen Liu, Xu Feng

**Affiliations:** Collage of Management and Economics, Tianjin University, Tianjin, China

**Keywords:** Agent-based modeling, Strategic communication, Searching behavior, Information identification

## Abstract

We build an agent-based cheap talk communication model with dynamic information searching behavior. In this model, agent communicates with its neighbors freely to get true or false information. Moreover, the uninformed receivers has ability to break up one link of his neighbor who is considered as a dishonest sender and searches for a new neighbor in the market. We study the impacts of the accuracy of information, the number of neighbors and the percentage of relinking neighbors on the information identification of uninformed receivers. The results suggest that the effect of the accuracy of information and the number of neighbors on information identification is linear, but the effect of the percentage of relinking neighbors presents a first increasing and then decreasing trend.

## Background

In the model of cheap talk communication, uninformed agents (receivers) receive possibly noisy messages from informed agents (senders) who are possibly dishonest. Talk is cheap because the communication between the two types of agents does not directly affect the payoffs regarding the messages both agents obtain from the game. The way the messages matter is by changing the receiver’s belief about the sender’s type, the messages can change the receiver’s action, which affects both agents’ payoffs indirectly.

The study of cheap talk models was motivated by the inefficiency resulted from asymmetric information. As noted by Sobel (2013), with differences in information, agents can improve their outcomes with direct, costless communication. However, the information cannot be transmitted faithfully when information sender has conflict of interest. The receiver has to identify whether the information came from costless communication is creditable or not. In the past few years, the cheap talk communication has been extended to multiple agents and different kinds of communicating networks, like Krishna and Morgan (2001), Ambrus and Takahashi (2008), Klumpp (2007) and Li (2010). These models are based on the assumption that the uninformed receiver gets information from his neighbors who has fixed links with him. The information dissemination network was established from the initial period and would not be changed during the game. Uninformed receivers have fixed information sources and would not search a more reliable information source. Difference with these previous studies, Our study establishes an agent-based model (Schoham and Leyton-Brown 2008) to describe dynamic information searching behavior of uninformed receivers. Receiver not only communicates with his neighbors, but also searches new information source to take the place of the old neighbor who he thinks is the least reliable one. The searching behavior in our model is more conform to reality, and helps us to understand whether the asymmetric information decreases if uninformed receivers has probability to find better information sources, which is not be addressed in the previous studies.

The model established in our study is not only based on theory and computer simulation, but also based on real life examples provided by available literatures. One example is about the cheap talk communication in the real life. Lots of such kinds of communication examples existed on Internet. With stock messages board, twitter or blogs, information of listed companies is transmitted among individuals freely. However, the information on Internet may be not accurate. Some scholars have conducted studies about this phenomenon. As Depken and Zhang (2010) pointed, the messages issued through Internet messages board by authors are inherently a world of cheap talk. Their study suggested that high-reputation authors tend to offer accurate information strategically. Another example is about agents learning process. In real-world situations, individuals make decisions based on partial inputting information (experience) rather than fully rational. They will take certain learning behavior, like reinforcement learning, to making decision. Gan et al. (2012) organized experiments to study the information searching function of academic users. They found that the reinforcement learning models can appropriately fit the information searching process. Wang et al. (2014) employed a Rock-Paper-Scissors game experiment to test how individuals make decisions strategically. They also find that reinforcement learning model can be used for describing the decision making of individuals.

We test the impact of the accuracy of information transmission, the number of neighbors and the percentage of relinking neighbors on the information identification of uninformed receivers. Our study finds that effect of the accuracy of information and the number of neighbors on information identification is positively related with the information identification of uninformed receivers, but the effect of the number of relinking neighbors first shows a first increasing and then decreasing trend, which implicates that a moderate relinking number (searching behavior) might maximally eliminate the asymmetric information between information senders and receivers.

## Literature review

The initial cheap talk communications focused on characterizing the behaviors and strategies of individuals and continued the basic communication framework of one sender and one receiver, as in Crawford and Sobel (1982) and Crawford (1998). These studies focused on the strategic communications with rational behavior in which an informed sender who is honest or dishonest sends a signal to the receivers, who take actions to identify whether the sender is honest or not. The model explained how information is strategically transmitted when agents have partially aligned interests. Lots of studies Crawford and Sobel (1982), Blume et al. (2007), Galeotti et al. (2013), Benabou and Laroque (1988) and Farrel (1995) extended the initial communication framework and found that the accuracy of information, number of agents (senders or receivers), and communication network are important factors for the identification ability of uninformed receivers.

Accuracy of information is important because the honest senders might be identified as the dishonest one if he observed false information and delivered it to receivers honestly. Benabou and Laroque (1992) modeled such kind of communication in a stock market. In the Benabou and Laroque (1992), senders had motivation to manipulate stock price by releasing noisy messages. Receivers can not distinguish the honest sender in the long run if the information is inaccurate and the dishonest sender change his communication strategy stochastically. Blume et al. (2007) found that adding an optimal noise level into the communicating information might improve the welfare of agents. Ben-Porath (2003) considered the scenario of pre-play communication. Chakraborty and Harbaugh (2007) and Chen (2011) made more assumption on the accuracy of information and the behavior of uninformed receivers.

There also has been a growing number of studies focusing on cheap talk communications with multiple senders and receivers. Krishna and Morgan (2001) built a model with two experts who have the same or opposite biases and communicate with receivers sequentially. In studies by Battaglini (2002) and Ambrus and Takahashi (2008), the senders can observe the real states in all dimensions and their decisions are two-dimensional variables. Klumpp (2007) introduced a sufficiently large number of informed senders and found that the information will be truthfully revealed to the uninformed receivers. Moreover, Klumpp (2007) drawn conclusion that as the number of informed traders increases, the estimations of firm value converges in probability, even though the informed traders retain some of their private information. Li (2010) studied a model with two experts observing a perfect reality but each of them has bias in his own private information.

The network structure of information transmission became another important topic in the recent strategic communication studies. Hagenbach and Koessler (2010) considered a network of strategic communication. They found that the connected network of agents has impact on the equilibrium of the model. In their model, every agent would like to take an action that is coordinated with those of others. An agent would reveal his information to a group which is large enough, and his ideal action should be close to the average action of the other agents in that group. Galeotti et al. (2013) introduced a model that agent only sends messages to the agents who has a fixed link with him. They found that the equilibrium not only depends on the conflict of interests between the agents, but also on the network structures of the model. Agastya et al. (2015) investigated situations in which agents communicate with each other through a chain of intermediators and found that the loss of information relative to direct communication is proportional to the number of intermediators involved in the chain.

Our paper consider the network of communications as a dynamic one rather than a static one. Also, we consider the impact of accuracy of information and the number of agents on the identification of receivers under the condition of dynamic network. Our study is related with previous studies. We found that the accuracy of information and the number of neighbors positively related with the identification ability of receivers, which is consistent with the Benabou and Laroque (1992) and the Krishna and Morgan (2001) or Klumpp (2007). Moreover, our paper made more considerations on the dynamic searching behavior in the cheap talk communication. We not only reach conclusions of available literatures about the influence of number of neighbors and information accuracy on identification, but also found relationships between the dynamic information communication network and identification ability, which are the previous models did not give a explicit description.

## The model

Consider a communication in which the Sender $S_i$ (the informed agents, $i = 1, \ldots ,N$) possesses private information about different issues that the Receiver $R_j$ (the uninformed agents, $j = 1, \ldots ,M$) takes actions on. The informed agents are further divided into two groups. One is the honest informed agents $S_h$, whose number is *h*, and who release the real information they receive, while the other is dishonest informed agents $S_d$, the quantity of which is *d*, who release real or false information.

Uninformed agents only receive messages from their neighbors and do not share information with each other. After sorting uninformed agents by current payoffs in every period, some of the uninformed agents at the lowest ranked locations will reselect their information sources, thus cutting off the informed agent he trusts the least and searching for another informed agent who is not directly connected to him in the current communication, thereby excluding the informed agent at the lowest ranked location in this period.

### Decision-making and prediction of individuals

At any period *t* ($t = 1, \ldots ,P$) the real state of information is $m_t$, which is a random sequence that follows a normal distribution *N*(0, 1). Information ${m_{i,t}}$ that the *i*th Sender receives at the same time is

1 $$\begin{aligned} {m_{i,t}} = {m_t} + b \cdot \gamma \end{aligned}$$

where $b \cdot \gamma$ is the bias Sender *i* gets at period *t* from the real state, $b \sim N(0,1),\,\gamma$ is the baseline of accuracy, which is a scalar parameter that we will use later to measure the accuracy of the information, such that the smaller that $\gamma$ is, the higher the accuracy of the information the Sender receives is.

In our model, honest Senders would send the information as they receive it, ${m_{i,t}}$, at period *t*, while the dishonest Senders probably send noisy messages to Receivers. We define noise using the Sigmoid function, which is a strictly increasing function having an“S” shape. We use a sigmoid function to model the relationship between noise and expectation is because that sigmoid function is a strictly increasing function. This function could reflect that higher expected payoffs the dishonest agents would like to get, the more noise he will release. In addition, S shaped also inflects the marginal decrease of noise that an informed agent sends to his neighbors. As the increase on expectation of informed agent, he will send larger noise. However, a larger noise is hardly accepted by the unformed agents, and will lead more decrease on informed agents reputation. Therefore, we set a strictly increasing but marginal decrease function to describe the telling lies behavior of informed agents. Thus, for each dishonest Sender, the noise they will release is

2 $$\begin{aligned} {n_{i,t}} = {\left( { - 1} \right) ^{{x_{i,t}}}}\left( {\frac{1}{{1 + \exp \left( { - {E_{i,t}}} \right) }} - 1} \right) \end{aligned}$$

where ${x_{i,t}}$ is a pseudorandom integer equaling 0 or 1.

Thus, the information the dishonest Sender releases is

3 $$\begin{aligned} {m_{id,t}} = {m_{i,t}} + {n_{i,t}}. \end{aligned}$$

The payoffs depend on the difference between ${m_{i,t}}$, the real states of information and ${y_{j,t}}$, the action uninformed receiver *j* takes. When Receivers (uninformed agents) receive messages from the informed Senders whom they connects with at period *t*, they may accept or reject the messages depending on their strategies, which we will discuss in detail in the following sections. When uninformed receiver *j* chooses to trust the information received from informed sender *i* at period *t*, we define parameter ${y_{j,t}}$ as equal to the information he received, that is, ${m_{i,t}}$ (if the informed sender is honest) or ${m_{i,t}} + {n_{i,t}}$ (if the informed sender is dishonest). While if he does not trust the information received, parameter ${y_{j,t}}$ equals 0, which depends on mean of information real states of 1. So payoff of uninformed receiver *j* obtains at period *t* is

4 $$\begin{aligned} {\pi _{j,t}} = - {\left( {y_{j,t} - m_{t} } \right) ^2}. \end{aligned}$$

Because the actions of Receiver decide the payoffs of both sides, the payoff of informed senders depend on how many uninformed receivers choose to trust him in the period. Only uninformed receivers linked with him trust the information he released, he could obtain payoff from this communication. So, payoff of informed receiver *i* obtains at period *t* is

5 $$\begin{aligned} {\pi _{i,t}} = \sum \limits _{j=1}^{M}{ - {\left( {y_{j,t} - m_{t} } \right) ^2} }. \end{aligned}$$

### Strategy updates of individuals

In this section, we describe the informed and uninformed agents’ strategies when they make decisions in a typical period, which we will discuss below.

#### Strategies of informed agents

Information released by the honest and dishonest informed agents is given by Eqs. (1) and (3), respectively. The honest informed agents have only one strategy, to release the real information they receive. For the dishonest informed agents, they have a choice in every period to update their strategy and decide whether to release noisy information. In this paper, we follow the work of Borgers and Sarin (2000) and assume that dishonest informed agents update their strategies with a learning process. The learning process assumes that agents have certain expectations of his chosen strategy. If the payoff brought by the current strategy can satisfies his expectations, he will increase his probability of current strategy in the next period, otherwise he will change his strategy.

The learning process is a Markovian process. Whether an agent will be more likely to undertake an action at period $t+1$ only depends on the payoff he receives at period *t*. At any period *t*, a dishonest informed agent *i* adopts a strategy ${s_{i,t}}$ with an expected payoff ${E_{i,t}}$, but at period *t*, he is actually paid $\pi _{i,t}$. Thus, at period $t+1$ the probability ${p_i}\left( {t + 1} \right)$ of the agent lying can be expressed as:

If the agent lies at period *t*,

6 $$\begin{aligned} {p_i}\left( {t + 1} \right) = \left\{ \begin{array}{ll} {p_i}\left( t \right) + \alpha \cdot \left( {1 - {p_i}\left( t \right) } \right) ,&{}\quad {\pi _{i,t}} \ge {E_{i,t}}\\ {p_i}\left( t \right) - \alpha \cdot {p_i}\left( t \right) ,&{}\quad {\pi _{i,t}} < {E_{i,t}} \end{array} \right. \end{aligned}$$

If the agent does not lie at period *t*,

7 $$\begin{aligned} {p_i}\left( {t + 1} \right) = \left\{ \begin{array}{ll} {p_i}\left( t \right) - \alpha \cdot {p_i}\left( t \right) ,&{}\quad {\pi _{i,t}} \ge {E_{i,t}}\\ {p_i}\left( t \right) + \alpha \cdot \left( {1 - {p_i}\left( t \right) } \right) ,&{}\quad {\pi _{i,t}} < {E_{i,t}} \end{array} \right. \end{aligned}$$

where the agent’s payoff at period $t+1$ is ${E_{i,t + 1}} = \beta \cdot {\pi _{i,t}} + \left( {1 - \beta } \right) \cdot {E_{i,t}}$.

${E_{i,t}}$ represents the expected payoff of an individual regarding his current strategy. With an exogenous aspiration level, if the actual payoff coming after strategy ${s_{i,t}}$ is above some threshold ${E_{i,t}}$, the *i*th agent would be satisfied with his current strategy, and therefore he tends to choose ${s_{i,t}}$ in the next period, and vice versa. ${E_{i,t+1}}$ reveals an agent’s endogenous expected payoff at period $t+1$, which shows that the expected payoff in the next period will be between the expected payoff and actual return from this period. $\alpha$ is the rate of selected strategy adjustment, and $\beta$ is the rate of adjustment of ${E_{i,t+1}}$, both of which are fixed values in our model between 0 and 1.

#### Strategies of uninformed agents

Uninformed agents also adopt the learning process to update the credibility of the informed agents connected to them in the communication. Presume that ${b_{i,j,t}}$ is the confidence of the *j*th uninformed agent in the *i*th informed agent at the *t* information transmission period. Whether the uninformed agent trusts the informed agent depends on the credibility ${b_{i,j,t}}$ of all of the informed agents connected to him. At one period, one uninformed agent may receive multiple messages from adjacent informed agents in the process of information transmission. We introduce a mechanism of competition with the aim that an uninformed agent can choose a message released by an informed agent in whom he has the highest confidence and update the confidence parameter ${b_{i,j,t}}$ for every informed agent connected to him based on the information from these sources.

We suppose an uninformed agent can only choose a message released from one of the information sources in whom he has the highest confidence. In addition, each uninformed agent records all of the messages passed to him. When he receives the actual states ${m_{t + 1}}$ in period $t+1$, the uninformed agents will examine whether the informed agents have ever lied and make an adjustment regarding ${b_{i,j,t + 1}}$ their confidence in them in the next period. The method of adjustment is as follows:

8 $$\begin{aligned} {b_{i,j,t + 1}} = \left\{ \begin{array}{ll} {b_{i,j,t}} + \left( {1 - {b_{i,j,t}}} \right) \cdot \theta ,&{}\quad {\mu _{i,j,t}} \le {{\bar{\mathrm{M}}}_{i,j,t}}\\ {b_{i,j,t}} - {b_{i,j,t}} \cdot \theta ,&{}\quad {\mu _{i,j,t}} > {{\bar{\mathrm{M}}}_{i,j,t}} \end{array} \right. \end{aligned}$$

where ${\mu _{i,j,t}}$ is the bias of information an uninformed agent *j* receives from the informed agent *i* connected to him ${m_{i,j,t}}$ and the real state ${m_t}$. ${\bar{\mathrm{M}}_{i,j,t}} = \frac{1}{M \cdot k}\sum \nolimits _i {\sum \limits _j {{\mu _{i,j,t}}} }$ is an average value of all of the information biases that all of the uninformed agents receive from their informed neighbors with the real state at period *t*. $\theta$ is the rate of confidence adjustment of uninformed agents to informed agents, which is a fixed number between 0 and 1. When the information the *i*th informed agent provides to the *j*th uninformed agent is more precise than the average, ${\mu _{i,j,t}} \le {{\bar{\mathrm{M}}}_{i,j,t}}$, the *j*th uninformed agent will increase his confidence in the *i*th informed agent, and vice versa.

### Searching behavior

In the information network construction, we assume each uninformed agent has *k* informed neighbors. In this network, only informed agents can become an information source. If the number of informed Senders $i \ge 2$, there is more than one source of information in the network.

Among informed agents, they do not convey messages to each other, they only release the messages to the uninformed agents who are linked to them. The uninformed agents can only receive messages from the informed agents who are linked to them, but they do not know whether the informed agents are honest. When they receive messages from informed agents in any period, they have the choice to believe one of the informed agent, which depends on the parameter called confidence in informed agents that is determined by the confidence one uninformed agent has in one informed agent based on the actions both have already taken. If an uninformed agent believes in one informed agent, the uninformed agent has the highest confidence, the uninformed agent will receive a messages from this informed agent and believe that it is true. Under the effect of this competitive atmosphere among informed agents, uninformed agents will preserve all of the messages released to them so they can update their confidence level in the informed agents who are adjacent to them. The informed agents also will be aware of how many uninformed agent accept his message. In each period, the payoff of one informed agents is the superposition of each payoff he gets from the uninformed agents he linked with. From the payoffs, an informed agent could learn whether the uninformed agents he linked with thinks he is honest or not. This is an endogenous and dynamic learning process that the informed adjust strategies through the increased or decreased payoffs.

At the end of each period, some of uninformed agents update their information sources by cutting off their connection with one of the informed agents linked to them in whom they have the lowest confidence. The uninformed agent will find a new information source to fill the vacancy. We define $\Gamma$ as the percentage of relinks in each period. That is, after sorting uninformed agents by current payoffs in every period, $\Gamma$ percent of uninformed agents at the lowest ranked locations will reselect their information sources. Suppose the *j*th uninformed agent is the agent who needs to take this action. The uninformed agent sorts the *k* informed agents who connect with him based on how well he trusts them (the parameter ${b_{i,j,t}}$ that we introduced above), and then he cuts off the informed agent $k^{\prime }$ whom he trusts the least and searches for another informed agent who is not directly connected to him in the network, but excluding informed agent $k^{\prime }$. By doing so, the uninformed agents can improve their searching behavior to receive more true messages as time passes. To simplify our modeling, there is no cost to identifying the level of confidence in information sources, and uninformed agents select a new source of information to reconnect with at random and reset their trust, thus giving an initial value of 0.5 to their confidence in the newly linked informed agent.

**Fig. 1:**
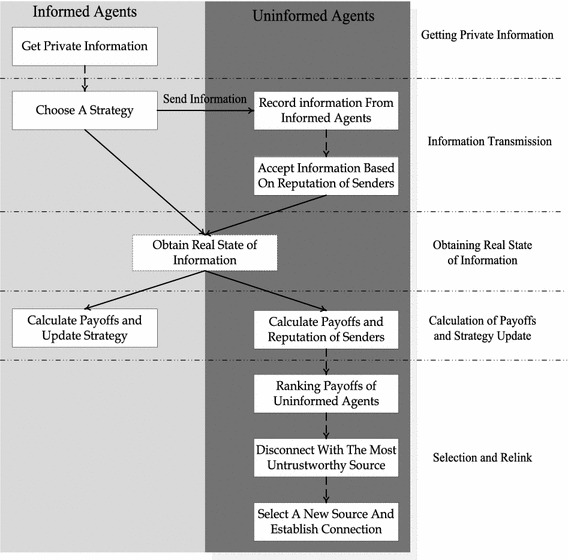
Flow of the information transmission process

A typical information transmission process is shown in Fig. 1.

## The simulation settings

The model requires the specification of a number of parameters that are summarized in Table 1 and discussed below.

**Table 1:** Model parameters

		Base case
*N*	Number of informed agents	100
*M*	Number of uninformed agents	900
*I*	Percentage of honest informed agents	50
$\Gamma$	Number of uninformed agents with searching behavior	0, 10, 20, 30, 100, 300, 900
*k*	Number of neighbors of uninformed agents	2, 4, 6, 8, 10
*P*	Total number of periods	5000
$m_p$	Real states of information	U[0, 1]
${x_{i,p}}$	Pseudorandom integer	0, 1
*b*	An array of random numbers from a normal distribution	N[0, 1]
$\gamma$	Scalar parameter measuring accuracy of the information	0.1, 0.3, 0.5, 0.7, 0.9
$\alpha$	Rate of selected strategy adjustment	0.05
$\beta$	Rate of adjustment of expected payoff	0.9
$\theta$	Rate of trustworthiness adjustment	0.05

In the baseline model, we have considered two types of agents, 900 uninformed agents and 100 informed agents, who are further divided into two groups, an honest group and a dishonest group, both of which account for 50 % of the total. The simulations consist of a number of information transmission periods $P=5000$. Information on real states in every period is randomly generated from a normal distribution *N* (0, 1). At the beginning of the information transmission, informed agents (honest and dishonest) obtain accurate or inaccurate information with a certain probability, and then dishonest informed agents select effective strategies based on their actual income from the prior periods. We set scalar parameter $\gamma$ to measure the accuracy of the information as 0.1, 0.3, 0.5, 0.7, 0.9 with the aim of examining the impact of the accuracy of the information. The smaller $\gamma$ that is, the more precise information the informed agents will receive.

The simulations draw the information searching is set as follows. Not all uninformed agents find new information sources every period. Based on a wealth-driven mechanism, all of the uninformed agents are arranged in descending order of the ranks of their payoffs from the communication, and only the bottom $\Gamma$ percent of uninformed agents face under economic incentive and look for better sources of information. Our model differs from that of others because our uninformed agents have searching behavior. This article will detect the impact of searching capabilities on the information identification of uninformed agents.

In the information transmission, every uninformed agent is linked with *k* informed agents, who are what we call neighbors. In our simulation, to ensure that uninformed agents have enough optional information sources and to verify the influence of the number of information sources on the identifications made by uninformed agents, *k* is assigned as 2, 4, 6, 8, 10.

## Simulation analysis

In this section we analyze, via numerical simulations, the factors that influence information identification implied by the model described in “The model” section. The results are outlined and discussed in this section.

The results reported here are the outcomes of simulations of 5000 periods, each of which we repeat 30 times using different random seeds for the random number generators. To test the influences on the information identification of the uninformed agents, we have repeated the simulations by varying the parameters sets of the accuracy of information, the number of neighbors and the number of uninformed agents with searching behavior. Because the number of honest informed agents that uninformed agents choose to trust fully represents the informed agents’ identification, we mainly focus on these results instead of the other statistics.

### Influence of variance of individual information sources

Our main aim in this section is to gain insights into the factors that influence uninformed agents’ information identification in the model. Some of the parameter settings for our simulations are presented in the previous section. In the base case, honest and dishonest informed agents are a fifty-fifty proportion, and the parameter $\gamma$ measuring their information accuracy is 0.5, which typifies a relatively higher level of precision. In every period, the percentage of uninformed agents with searching behavior who will find a new neighbor with whom to relink will be $\Gamma =10$. Under these settings, the number of neighbors in the different simulations ranged from 2 to 10.

**Fig. 2:**
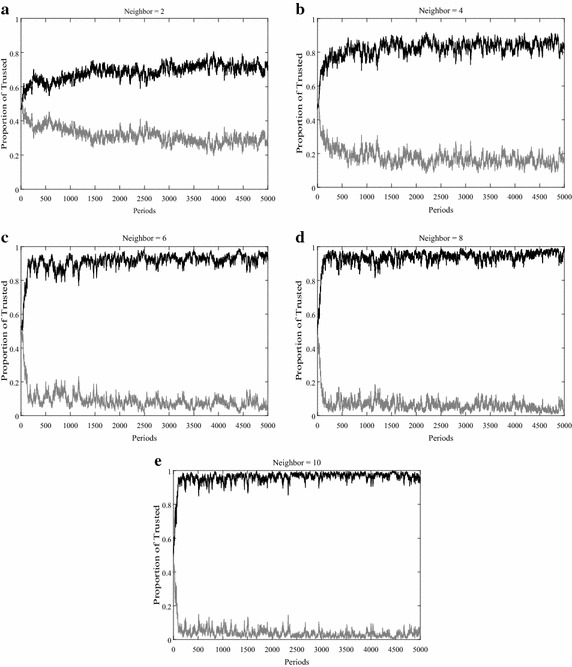
Proportions of the trusted honest agents and trusted dishonest agents. Proportions of honest agents (*black*) and dishonest informed agents (*grey*) that the uninformed agents choose to trust over time with the accuracy of information set at 0.5, the percentage of relink set at 10, and the uninformed agents have numbers of neighbors which equal to 2, 4, 6, 8, 10 in subfigure **a** to **e** respectively

The number of honest informed agents that uninformed agents choose to trust over time reveals the information identification ability of the uninformed agents observed in the simulations. Figure 2 contrasts the proportions of honest and dishonest informed agents that the uninformed agents choose to trust over time. Uninformed agents fail to distinguish honest agents from dishonest agents at the beginning, but as time goes by, uninformed agents begin to discern the honest agents in the first 500–1000 periods, and the proportion of honest agents starts to rise, while the proportion of dishonest agents falls. The proportion of honest agents then remains relatively stable at a high level after growing sharply. Taking into account the changes in the number of neighbors, the changes in information identification ability coincide with the increase in the number of neighbors of the uninformed agents, thus showing an approximately positive and monotonous relationship. When the uninformed agents have fewer neighbors in their network, <70 % of the information they use is obtained from honest agents, and when the number of neighbors climbs to 10, more than 96 % of the information that the uninformed agents receive is released by honest agents. Keeping the number of honest and dishonest informed agents constant, the fewer neighbors the uninformed agents have, the more noisy messages that are spread through individuals in the communication, while the more neighbors the uninformed agents have, the more that truthful information is widely disseminated. This result can be explained as when uninformed agents has more neighbors, they have more choices to select one information source to trust in, which is released by the informed agent with highest reputation. So more neighbors lead to more choices when one uninformed makes decisions and with valuing reputation, to make optimal decision. These conclusions consistent with the previous studies of Crawford and Sobel (1982), Blume et al. (2007), Galeotti et al. (2013) and Hagenbach and Koessler (2010).

It can be observed from the comparison that, with an increasing quantity of neighbors, the ability of the uninformed agents to identify information improves. The more neighbors that one uninformed agent has, the earlier that agent will reach an equilibrium. Figure 2 shows that when one uninformed agent has only 2 neighbors, it takes him 1000 periods or even longer to achieve an equilibrium to identify honest informed agents from the dishonest agents, while when an uninformed agent has 6 or more neighbors, he can quickly reach equilibrium. If an uninformed agent has more information sources, he can identify the honest sources more efficiently and precisely through the confidence parameter.

Table 2 shows the means and coefficients of the variations (C.V., also known as relative standard deviation, calculated as the ratio of the standard deviation to the mean) of the proportion of honest informed agents under different conditions of quantity of neighbors ranging from 2 to 10. The average proportion of honest informed agents that the uninformed agents choose to believe in under the equilibrium states reflects, to a degree, the identification capabilities of the uninformed agents and illustrates the impact of noise on information transmission. The C.V. of the proportions reveal the stability of the agents’ identification ability. From Table 2, we can see that, when the parameter $\gamma$, which measures the accuracy of the information the informed agents receive, is set at 0.5, the C.V. decreases with a varying number of neighbors. If we change the value of parameter $\gamma$, we find that the accuracy of the information has something to do with the identification ability of the uninformed agents. Comparing the statistics from Tables 3 and 4, we can conclude that when the informed agents receive relatively accurate information from the model, the C.V. of the proportion decreases as the quantity of neighbors increases, but when the information captured within the model lacks sufficient precision, the C.V. increases progressively. The reason for this result can be explained as follows. When information accuracy is relative high, more neighbors one uninformed has, more choices he has to select an information source with highest reputation. Under conditions with low information accuracy, the information uninformed receives is far from the real state of information. Even dishonest agents release noise to uninformed agents, it is not very easy for uninformed agents to tell the difference between noise and inaccuracy of information, that made uninformed agents confused to identify. Further more, increasing number of neighbors may add to confusion during information identifying process. More neighbors (more information sources) may increase volatility of identification capability. This result displays that information accuracy has a greater influence on identification. These conclusions consistent with the previous studies of Blume et al. (2007) and Farrel (1995).

**Table 2:** The means and coefficients of the variations of the proportions of honest informed agents with $\gamma$ equals 0.5

	$k=2$ (%)	$k=4$ (%)	$k=6$ (%)	$k=8$ (%)	$k=10$ (%)
Mean	70.75	84.06	92.81	94.64	96.97
C.V.	4.38	3.63	2.58	2.38	1.71

The means and coefficients of the variations of the proportions of honest informed agents when the number of neighbors of the uninformed agents separately equal 2, 4, 6, 8, 10, and the percentage of relinks equals 10

We believe that the trends shown in Table 3 suggest that if the accuracy of information is high, the information that honest informed agents receive and release is close to the real state, so the profits of both sides would be satisfactory. Thus, the uniformed agents could easily establish confidence in the honest agents with whom they are linked, i.e. the confidence parameter ${b_{i,j,t}}$ is high, and most of the uninformed agents drive out the dishonest senders of information by their searching behavior. If one uniformed agent has more neighbors, it means that he has access to more information sources and chooses the one he is most confident in. A similar analysis can be made in Table 4, in which the accuracy of information is low. In this case, the information the informed agents receive is quite different from the real state, no matter whether the informed agent is honest, and the information that the uninformed agents receive from the informed agents may be far from the truth. Thus, the informed agents’ reputation endowed by the uninformed, which is used to reflect the reliability of the resources is relatively poor, even when one informed agent is honest and sends messages in accordance with what he receives. If the uninformed agents have more neighbors, there are more chances for the spreading of noise in the communications. Noise transmission interferes with the selection of the uninformed agents.

**Table 3:** The means and coefficients of the variations of the proportions of honest informed agents with $\gamma$ equals 0.3

	$k=2$ (%)	$k=4$ (%)	$k=6$ (%)	$k=8$ (%)	$k=10$ (%)
Mean	99.14	99.91	99.99	100.00	100.00
C.V.	0.39	0.10	0.03	0.01	0.00

The means and coefficients of the variations of the proportions of honest informed agents when the number of neighbors of the uninformed agents separately equal 2, 4, 6, 8, 10, and the percentage of relinks equals 10

**Table 4:** The means and coefficients of the variations of the proportions of honest informed agents with $\gamma$ equals 0.9

	$k=2$ (%)	$k=4$ (%)	$k=6$ (%)	$k=8$ (%)	$k=10$ (%)
Mean	49.83	57.81	63.55	66.75	70.31
C.V.	6.67	8.30	8.86	10.80	11.04

The means and coefficients of the variations of the proportions of honest informed agents when the number of neighbors of the uninformed agents separately equal 2, 4, 6, 8, 10, and the percentage of relinks equals 10

**Fig. 3:**
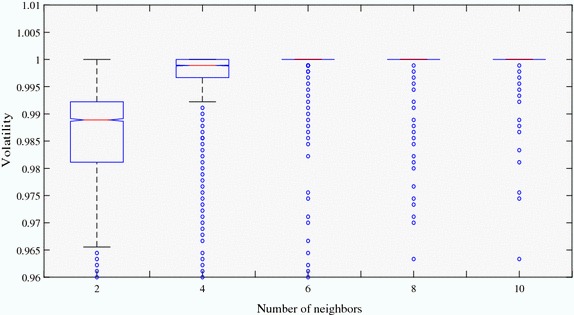
The changes in information identification by variances in the number of neighbors. The changes in uninformed agents’ information identification resulting from variances in the number of neighbors the uninformed agents have where the accuracy of information equals 0.3, and the percentage of relink is 10

Table 5 presents the results of T tests for the proportions of honest informed agents that the uninformed agents choose to trust with different quantities of neighbors. The p values, except those for groups of 6 and 8, and 8 and 10, shown in the table suggest that each two data sets with different numbers of neighbors are significantly different from each other. However, when the uninformed agents have enough neighbors, the impact of the changes in the quantity of neighbors on identification ability is mild. This finding is further corroborated by Fig. 3. Under the condition that the index measuring information accuracy equals 0.3, fewer neighbors has a relatively strong impact on the stability of agents’ identification ability in comparison with having more neighbors.

**Table 5:** T test results for the proportions of trusted honest informed agents with different quantities of neighbors with whom the uninformed agents connected

Neighbor	2	4	6	8	10
2	–	7.3308E−096	2.6701E−125	1.7400E−151	1.0429E−179
4	7.3308E−96	–	1.3457E−004	7.9350E−009	4.0679E−015
6	2.6701E−125	1.3457E−004	–	0.1017	7.3103E−004
8	1.7400E−151	7.9350E−009	0.1017	–	0.0709
10	1.0429E−179	4.0679E−015	7.3101E−004	0.0709	–

Significant at the 1 % critical level using a two-tail test

p value of the T test result for the proportions of trusted honest informed agents with different quantities of neighbors with whom the uninformed agents connected. The accuracy of information is 0.3, and the percentage of relinks equals 10

### Influence of information accuracy

The accuracy of the information informed agents receive has a significant impact on the identification abilities of the uninformed agents. In this new case, we maintain the same network structure, we set 4 neighbors for every uninformed agent and we make an equal split between honest and dishonest informed agents. In every period, there are 10 % of uninformed agents who will find a new neighbor with whom to link. Under these conditions, we change the parameter of the accuracy of the information, $\gamma$, in different simulations ranging from 0.1 to 0.9. Lower values mean that the information is more accurate.

**Fig. 4:**
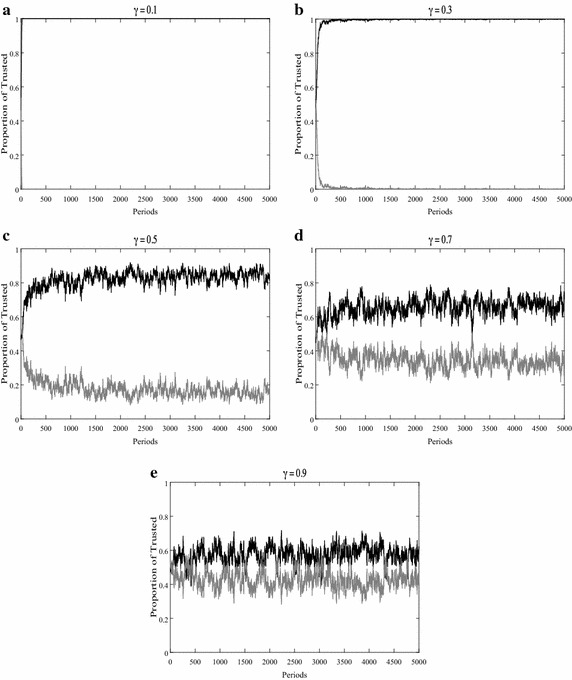
Proportions of the trusted honest agents and trusted dishonest agents. Proportions of honest informed agents (*black*) and dishonest informed agents (*grey*) whom the uninformed agents choose to trust over time with the number of neighbors set at 4 and the percentage of relink set at 10. The accuracy of the information changes with value 0.1, 0.3, 0.5, 0.7, 0.9 in subfigure **a** to **e** respectively

Figure 4 illustrates what happens to the information identification of the uninformed agents as the information accuracy changes. We find that the effect of information accuracy on identification is positive and monotonous. When the informed agents receive precise information in the simulations, the uninformed agents can pick out the honest agents from the dishonest agents rapidly and accurately. If the information is inaccurate, the identification ability plummets, especially when the parameter $\gamma$ equals 0.9, when the uninformed agents can hardly distinguish the honest agents from the dishonest agents from the beginning of the simulations to the end. Under this circumstance, the uninformed agents are paid less and information the uninformed agents receive from their honest neighbors is not much different from that received from dishonest neighbors, even though the honest agents never lies.

Table 6 reveals the obvious relationship between information accuracy and information identification. C.V., which represents the stability of agents’ identification capabilities, increases as parameter $\gamma$ increases. Highly accurate information helps the uninformed agents make a distinction between the honest and the dishonest informed agents, this result is the same as our intuition.

**Table 6:** The means and coefficients of the variations of the proportions of honest informed agents

	$\gamma =0.1$ (%)	$\gamma =0.3$ (%)	$\gamma =0.5$ (%)	$\gamma =0.7$ (%)	$\gamma =0.9$ (%)
Mean	100.00	99.91	84.06	66.79	57.81
C.V.	0.00	0.10	3.63	6.44	8.30

The means and coefficients of the variations of the proportions of honest informed agents when the accuracy of the information separately equals 0.1, 0.3, 0.5, 0.7, 0.9, the number of neighbors one uninformed agent has equals 4, and the percentage of relinks is 10

**Fig. 5:**
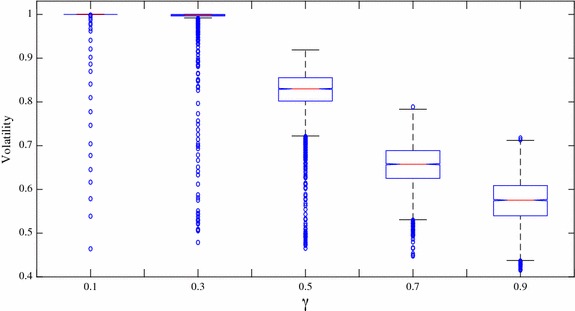
The changes in information identification by variances in information accuracy. The changes in the uninformed agents’ information identification resulting from variances in the accuracy of information when the number of neighbors that one uninformed agent has equals 4, and the percentage of relink is 10

The T tests for information accuracy were also checked and the results are shown in Table 7. The p values shown in the table, suggest that each two data sets with different information accuracy levels are significantly different from each other. This result indicates that the accuracy of the information that the informed agents receive plays an important role in the identification abilities of the uninformed agents when they choose information sources. Figure 5 also shows this difference.

**Table 7:** T test results for the proportions of trusted honest informed agents with different information accuracy

$\gamma$	0.1	0.3	0.5	0.7	0.9
0.1	–	2.1915E−23	0	0	0
0.3	2.1915E−23	–	0	0	0
0.5	0	0	–	0	0
0.7	0	0	0	–	0
0.9	0	0	0	0	–

Significant at the 1 % critical level using a two-tail test

p value of T test result for the proportions of trusted honest informed agents with different information accuracy when the number of neighbors that one uninformed agent has equals 4, and the percentage of relinks equals 10

### Influence of the percentage of relinks

Varying both individual information sources and the accuracy of information can change the identification abilities of the uninformed agents. However, how does the searching behavior of the uninformed agents change? Some insight into this issue can be provided by analyzing the impact of the percentage of reconnects on information identification. In this section, we change the percentage of uninformed agents with searching behavior, that is, those who disconnect with one of their neighbors and find a new source with whom to reconnect, from $\Gamma =0, 10, 20, 30, \,\,\hbox {and}\,\, 100$. To eliminating the effect of information accuracy and the quantity of neighbors, we test the effect of the changes in the percentage of uninformed agents with searching behavior on identification with various levels of information accuracy and numbers of neighbors.

**Fig. 6:**
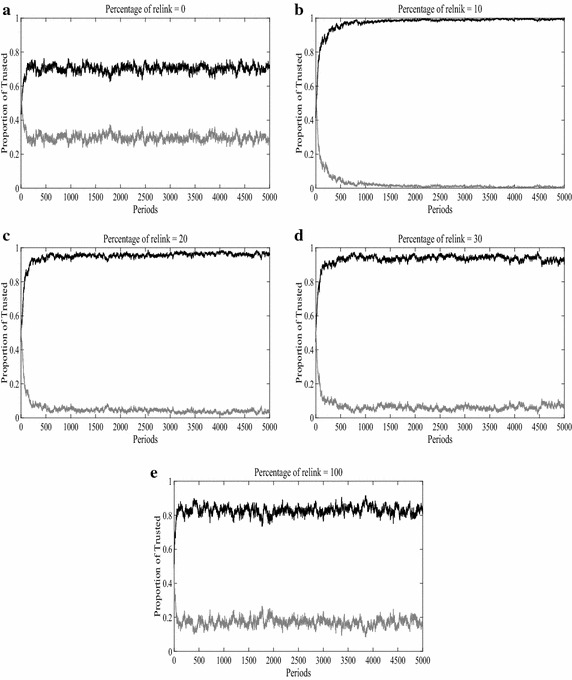
Proportions of the trusted honest agents and trusted dishonest agents. Proportions of honest informed agents (*black*) and dishonest informed agents (*grey*) that the uninformed agents choose to trust over time with the number of neighbors set at 2 and $\gamma$ set at 0.3. The percentage of relink changes with value 0, 10, 20, 30, 100 in subfigure **a** to **e** respectively

Figure 6 illustrates what happens to the information sources of the uninformed agents with different levels of searching behavior in the base case. As observed from the figures intuitively, the percentage of uninformed agents with searching behavior does not show a monotonous relationship with information identification that would affect the quality of information transmitted in communication, as the other two factors do. Viewed from the perspective of information sources and the quantity of neighbors, there are same trends in information identification with changes in the searching behavior of the uninformed agents in the simulation.

The average probability weights for the proportion of informed agents whom the uninformed agents trusts across the thirty base case runs are presented in Table 8. Under different conditions of information accuracy, the identification ability of the uninformed agents first increases and then decreases when the number of relinks increases. Under our parameter settings, when the percentage of relinks equals 10 (there are 10 % of uninformed agents that have the searching behavior), the information identification ability of the uninformed agents is strongest, and the stability of the agents’ identification capability is stable, while when the percentage of relinks is 100 (all of the uninformed agents search for new information sources), the uninformed agents have the lowest capability of information identification. That is, when the percentage of relinks is small, searching behavior leads to moderate improvements in information identification. However, when the percentage of relinks becomes large enough, searching behavior has a negative effect on the identification ability of the uninformed agents. The coefficients of the variances, which represent the degree of stability, have no obvious regularities.

**Table 8:** The means and coefficients of the variations of the proportions of honest informed agents with different percentages of relinks under different information accuracy levels

	$\Gamma =0$ (%)	$\Gamma =10$ (%)	$\Gamma =20$ (%)	$\Gamma =30$ (%)	$\Gamma =100$ (%)
$$\gamma =0.1$$					
Mean	100.00	100.00	100.00	100.00	100.00
C.V.	0.01	0.00	0.00	0.00	0.00
$$\gamma =0.3$$					
Mean	96.48	99.91	99.61	99.31	96.56
C.V.	0.97	0.10	0.27	0.38	1.00
$$\gamma =0.5$$					
Mean	77.27	84.06	77.36	75.30	70.15
C.V.	4.96	3.63	4.58	5.30	5.22
$$\gamma =0.7$$					
Mean	59.94	66.79	63.38	61.11	57.30
C.V.	7.15	6.44	7.43	7.49	7.94
$$\gamma =0.9$$					
Mean	55.55	57.81	55.83	52.92	54.36
C.V.	8.49	8.30	7.65	9.25	8.33

The means and coefficients of variations in the proportions of honest informed agents with different percentages of relinks under different information accuracy levels when the number of neighbors of the uninformed agents equals 4

A further test of the impact of the percentage of relinks on the information identification under situations with different numbers of neighbors is shown in Table 9. We reach a similar conclusion that identification ability first increases and then decreases. The values presented in Table 9 indicate that when 10, 20 or 30 % of uninformed agents have searching behavior, they have the strongest identification ability, and from this perspective, searching behavior leads to moderate improvements in information identification. As the percentage gradually increases, however, identification ability shows a downward trend, especially when $\Gamma$ equals 100, the identification ability of the uninformed agents is obviously reduced.

**Table 9:** The means and coefficients of the variations of the proportions of honest informed agents with different percentages of relinks under different numbers of neighbors

	$\Gamma =0$ (%)	$\Gamma =10$ (%)	$\Gamma =20$ (%)	$\Gamma =30$ (%)	$\Gamma =100$ (%)
$$k=2$$					
Mean	70.58	99.14	96.09	93.91	83.11
C.V.	2.68	0.39	0.96	1.45	2.79
$$k=4$$					
Mean	96.48	99.91	99.61	99.31	96.56
C.V.	0.97	0.10	0.27	0.38	1.00
$$k=6$$					
Mean	99.75	99.99	99.97	99.93	99.25
C.V.	0.23	0.03	0.06	0.10	0.42
$$k=8$$					
Mean	99.96	100.00	100.00	99.99	99.90
C.V.	0.08	0.01	0.02	0.03	0.13
$$k=10$$					
Mean	100.00	100.00	100.00	100.00	99.99
C.V.	0.01	0.00	0.00	0.01	0.04

The means and coefficients of the variations of the proportions of honest informed agents with different percentages of relinks under different numbers of neighbors when the accuracy of information equals 0.3

Instinctively, the identification ability should increase with searching behavior because the searching behavior that we introduced provides the agents with the ability to choose a new informed neighbor, which seems as if it should improve the uninformed agents’ identification performance. However, the results shown above do not match this assumption. To explain these results, we might consider in the real world, the decision to find a new informed neighbor may be based on economic inventive. At the end of each period, under our experimental setup, every uniformed agent is supposed to examine how well he performed in that period compared with other agents. If his payoff is less than the average payoff, he has an incentive to find a new informed neighbor, even if the new information source is dishonest and the old one is honest. As the number of relinks grows, more uninformed agents are exposed to the incentive, which makes the uninformed agents overreact or react inappropriately because of their relatively poorer payoffs.

Identification by the uninformed agents, on the condition that each uninformed agent has 4 neighbors and the parameter, $\gamma$, which measures information accuracy, equals 0.3, was tested and compared by T tests between groups. The results shown in Table 10 suggest that each group of two data sets with different percentages of relinks is significantly different. This result means that when the uninformed agents have 4 neighbors and the accuracy of information is set at 0.3, the quantity of uninformed agents with searching behavior has obvious significance to identification ability.

**Table 10:** T test results for the proportions of trusted honest informed agents with different percentages of relinks

$\Gamma$	0	10	20	30	100
0	–	0	0	0	1.3163E−004
10	0	–	2.0069E−005	8.2040E−015	0
20	0	2.0069E−005	–	4.1918E−004	0
30	0	8.2040E−015	4.1918E−004	–	0
100	1.3163E−004	0	0	0	–

Significant at the 1 % critical level using a two-tail test

p value of T test results for the proportions of trusted honest informed agents with different percentages of relinks when the number of neighbors that one uninformed agent has equals 4, and the accuracy of information equals 0.3

However, the results become different when the level of information accuracy changes. To examine the similarity of the data under the situations where the number of relinks is 10 and 100 in Table 8, a T test for the two groups was performed. The results are presented in Table 11. The p value is much different when the accuracy equals 0.1 from the other situations, which means that when information is extremely precisely transmitted in communication, the quantity of uninformed agents with searching behavior does not impact their identification ability. Table 12 displays the p value and the results of the T tests between the two groups with the percentage of relinks set at 0 and 100, and the results are similar to those shown in Table 9. The data for the two groups show statistical differences at a 1 % significance level. When the number of neighbors is high (equals 8 or more), the p value is much different from the other situations, which means that when uninformed has more neighbors, the percentage of uninformed agents with searching behavior does not impact their identification ability. But under other situations, the impacts of having no uninformed agents with searching behavior and all uninformed agents with searching behavior on information identification is quite different.

**Table 11:** T test results for the proportions of the trusted honest informed agents with the percentage of relinks set at 10 and 100

	$\gamma =0.1$	$\gamma =0.3$	$\gamma =0.5$	$\gamma =0.7$	$\gamma =0.9$
p value	0.5372	0	0	0	2.7504E−205

Significant at the 1 % critical level using a two-tail test

p value of T test results for the proportions of trusted honest informed agents with different information accuracy when the number of neighbors that one uninformed agent has equals 4

**Table 12:** T test results for the proportions of trusted honest informed agents with the percentage of relinks set at 0 and 100

	k = 2	k = 4	k = 6	k = 8	k = 10
p value	0	1.3163E−04	2.4366E−20	0.4438	0.5528

Significant at the 1 % critical level using a two-tail test

p value of T test results for the proportions of trusted honest informed agents with different numbers of neighbors that one uninformed agent has when the accuracy of information equals 0.3

## Robustness analysis

There are two constant ratios in the settings of agents, that is, 9:1 uninformed to informed ratio and 50:50 split on informed agents with honest and dishonest. To address the issue that whether these constant ratios will affect our results, we make robustness tests in this section. Specifically, we reset uninformed to informed ratio (Ru:Ri in Table 13) as 9:1, 5:5, 1:9, and reset honest to dishonest ratio (Rh:Rd in Table 14) as 3:7, 5:5, 7:3 to see whether our initial conclusions holds.

**Table 13:** The means and coefficients of the variations of the proportions of honest informed agents uninformed trusted with different uninformed to informed ratio

	$Neighbors=2$ (%)	$Neighbors=4$ (%)	$Neighbors=6$ (%)	$Neighbors=8$ (%)	$Neighbors=10$ (%)
$$Ru{:}Ri=9{:}1$$					
Mean	70.75	84.06	92.81	94.64	96.97
C.V.	4.38	3.63	2.58	2.38	1.71
$$Ru{:}Ri=5{:}5$$					
Mean	74.25	78.96	83.47	86.38	87.94
C.V.	4.11	3.80	4.04	3.79	3.72
$$Ru{:}Ri=1{:}9$$					
Mean	71.36	80.51	85.28	89.97	91.81
C.V.	6.36	5.43	5.04	4.07	4.12

	$\gamma =0.1$ (%)	$\gamma =0.3$ (%)	$\gamma =0.5$ (%)	$\gamma =0.7$ (%)	$\gamma =0.9$ (%)
$$Ru{:}Ri=9{:}1$$					
Mean	100.00	99.91	84.06	66.79	57.81
C.V.	0.00	0.10	3.63	6.44	8.30
$$Ru{:}Ri=5{:}5$$					
Mean	99.78	97.72	78.96	67.25	60.82
C.V.	1.90	2.55	3.80	4.59	4.78
$$Ru{:}Ri=1{:}9$$					
Mean	99.33	96.26	80.51	67.83	61.09
C.V.	1.80	3.38	5.43	7.06	8.54

	$relink=0$ (%)	$relink=10$ (%)	$relink=20$ (%)	$relink=30$ (%)	$relink=100$ (%)
$$Ru{:}Ri=9{:}1$$					
Mean	70.58	99.14	96.09	93.91	83.11
C.V.	2.68	0.39	0.96	1.45	2.79
$$Ru{:}Ri=5{:}5$$					
Mean	77.81	95.36	93.20	91.51	82.34
C.V.	2.88	3.16	2.91	2.90	2.96
$$Ru{:}Ri=1{:}9$$					
Mean	80.63	89.90	85.00	82.80	68.81
C.V.	4.48	4.00	4.89	5.22	7.69

1. Investigating the effect of the number of neighbors on information identification under different uninformed to informed ratios, other parameters are set as base case, honest and dishonest informed agents are a 55 proportion, the parameter measuring their information accuracy is 0.5, and percentage of relink is 10

2. Investigating the effect of information accuracy on information identification under different uninformed to informed ratios, other parameters are set as base case, honest and dishonest informed agents are a 55 proportion, the number of neighbors is 4, and percentage of relink is 10

3. Investigating the effect of percentage of relink on information identification under different uninformed to informed ratios, other parameters are set as base case, honest and dishonest informed agents are a 55 proportion, the number of neighbors is 4 and the parameter measuring their information accuracy is 0.5

**Table 14:** The means and coefficients of the variations of the proportions of honest informed agents uninformed trusted with different honest to dishonest ratio

	$Neighbors=2$ (%)	$Neighbors=4$ (%)	$Neighbors=6$ (%)	$Neighbors=8$ (%)	$Neighbors=10$ (%)
$$Rh{:}Rd=3{:}7$$					
Mean	35.81	40.87	42.85	46.43	49.91
C.V.	11.20	15.61	18.83	15.60	22.26
$$Rh{:}Rd=5{:}5$$					
Mean	70.75	84.06	92.81	94.64	96.97
C.V.	4.38	3.63	2.58	2.38	1.71
$$Rh{:}Rd=7{:}3$$					
Mean	77.52	81.89	84.56	84.40	85.98
C.V.	3.90	5.65	6.41	8.14	9.26

	$\gamma =0.1$ (%)	$\gamma =0.3$ (%)	$\gamma =0.5$ (%)	$\gamma =0.7$ (%)	$\gamma =0.9$ (%)
$$Rh{:}Rd=3{:}7$$					
Mean	97.52	54.42	40.87	34.40	32.76
C.V.	5.71	10.77	15.61	16.05	18.10
$$Rh{:}Rd=5{:}5$$					
Mean	100.00	99.91	84.06	66.79	57.81
C.V.	0.00	0.10	3.63	6.44	8.30
$$Rh{:}Rd=7{:}3$$					
Mean	99.50	91.64	81.89	73.03	75.34
C.V.	1.54	5.00	5.65	7.49	6.92

	$relink=0$ (%)	$relink=10$ (%)	$relink=20$ (%)	$relink=30$ (%)	$relink=100$ (%)
$$Rh{:}Rd=3{:}7$$					
Mean	35.15	50.41	42.78	41.74	39.62
C.V.	6.46	11.53	11.08	12.63	10.65
$$Rh{:}Rd=5{:}5$$					
Mean	70.58	99.14	96.09	93.91	83.11
C.V.	2.68	0.39	0.96	1.45	2.79
$$Rh{:}Rd=7{:}3$$					
Mean	76.75	88.76	83.03	84.52	80.30
C.V.	3.18	4.19	4.58	3.74	4.41

1. Investigating the effect of the number of neighbors on information identification under different honest to dishonest ratios, other parameters are set as base case, uninformed to informed ratio is 9:1, the parameter measuring their information accuracy is 0.5, and percentage of relink is 10

2. Investigating the effect of information accuracy on information identification under different honest to dishonest ratios, other parameters are set as base case, uninformed to informed ratio is 9:1, the number of neighbors is 4, and percentage of relink is 10

3. Investigating the effect of percentage of relink on information identification under different honest to dishonest ratios, other parameters are set as base case, uninformed to informed ratio is 9:1, the number of neighbors is 4 and the parameter measuring their information accuracy is 0.5

In order to measure the effect of uninformed to informed ratio on the number of neighbors, we set other parameters as same as base case, that is, honest and dishonest informed agents are a 55 proportion, the parameter $\gamma$ measuring their information accuracy is 0.5, and percentage of relink, $\Gamma$, is 10. Under these settings, the number of neighbors in the simulations ranged from 2 to 10 under different uninformed to informed ratios. We report the mean proportions of honest informed agents trust by uninformed agents over time and C.V. in Table 13.

Comparing data in the first part of Table 13, displays the robustness check for uninformed to informed ratio. Under different ratios, the ability of the uninformed agents to identify information improves with increasing quantities of neighbors. The result illustrates the information identification is robust to the number of neighbors with different uninformed to informed ratio.

To measure the impacts of uninformed to informed ratio on the accuracy of information, we set same parameters as base case, that is, 4 neighbors for every uninformed agent and an equal split between honest and dishonest informed agents. In every period, there are 10 % of uninformed agents who will find a new neighbor to link. Under these conditions, we change the parameter of the accuracy of the information, $\gamma$, ranging from 0.1 to 0.9, in simulations with different uninformed to informed ratios.

Comparing data in the second part of Table 13, though the volatilities of these figures are not exactly same, means and C.V.s under different ratios show the same tendency. The contrast among three sets of data profiles robustness of information identification to the number of neighbors with different uninformed to informed ratio.

In the same manner, we set other parameters as same as base case and vary the percent of relink to measure the robustness of information identification under different uninformed to informed ratios. Comparing data in the third part of Table 13, we learn that information identification does not show a monotonous relationship for the percentage of relink. The results remain presenting a first an increasing and then a decreasing trend.

Also we test the robustness of the honest to dishonest ratios on initial conclusions in Table 14. The parameters of the number of neighbors, the accuracy of information, the percentage of relink and the 9:1 uninformed to informed ratio are same as base case, which are also shown on previous simulations. We reset honest to dishonest ratio at 3:7 and 7:3 to exam the information identification trends present the same pattern with 5:5 honest to dishonest ratio of base case. Also, we report the mean proportions of honest informed agents trust by uninformed agents over time and C.V. in Table 14.

The effect of honest to dishonest ratio on the number of neighbors are presented in Table 14. Comparing data in the first part of Table 14, when the percentage of dishonest informed agents increases, the identification becomes more volatile. But the tendencies are same under different ratios. This could illustrate the information identification is robust to the number of neighbors with different honest to dishonest ratio.

Measurement results of the impacts of honest to dishonest ratio on the accuracy of information are shown in Table 14. Comparing data in the second part of Table 14, we learn robustness of information identification to the number of neighbors with different honest to dishonest ratio.

Similarly, we vary the percent of relink to measure the robustness of honest to dishonest ratio. Data in the third part of Table 14 present the same trend. When none possessing searching behavior, the information identification is relative low. As the percentage of relink gradually increases, identification ability shows a downward trend. When all of uninformed getting searching behavior, the identification ability is obviously reduced.

The comparison results in this section display the robustness check for effectiveness of information identification with two ratios, the uninformed to informed ratio and honest to dishonest ratio. This evidence demonstrates that the three factors, accuracy of information, the number of neighbors and percentage of relink, play significant roles in information identification of uninformed agents, while the number of uninformed and informed agents in population and the number of honest and dishonest in informed agents do not. In summary, the tests with certain assumptions here identify the three factors mainly affect information identification is reliable.

## Conclusions

In this paper, we presented a model of cheap talk communication while adding dynamic searching behavior for individuals. We set our model as a agent-based environment where informed players, either honest or dishonest, can transmit their information strategically to uninformed receivers who are connected with them in a communication network. In each period, the payoffs everyone receives and the reputations of the informed agents help receivers adjust their strategies accordingly. Moreover, receivers have ability to change one of his informed neighbors as into a new one in the market. We studied the impacts of the accuracy of information, the number of neighbors and the percentage of uninformed agents with searching behavior on the information identification of the uninformed agents.

Through agent-based modeling and data analysis, we found that an increasing number of neighbors has a positive influence on the uninformed agents’ information identification. Additionally, when the information the informed agents receive is relatively accurate, the stability of identification increases together with an increasing percentage of neighbors, but when the information captured within the model lacks sufficient precision, the stability progressively decreases. Another result is that the improvements in information accuracy can improve information selection and identification abilities. The stability of identification also changes with the information accuracy. Moreover, the third conclusion of this paper is that the dynamic searching behavior of receivers has nonlinear impact on their identification abilities. Under different conditions of information accuracy, the identification ability of uninformed agents shows a trend that first increases and then decreases when the percentage of relinks is increasing. When there are 10, 20 or 30 % uninformed receivers who has ability to search a new information sources in the market, the receivers group has the strongest identification ability.

Our model can be extended in several directions. For instance, models can be developed with individuals in a complex information environment searching for, obtaining and processing information in different ways, by focusing on individual behaviors, or by having uninformed agents send and receive messages from other uninformed agents to study how the equilibrium communication strategies change when the search scope of the uninformed agents varies.
